# Lagged meteorological impacts on COVID-19 incidence among high-risk counties in the United States—a spatiotemporal analysis

**DOI:** 10.1038/s41370-021-00356-y

**Published:** 2021-07-01

**Authors:** Lung-Chang Chien, L.-W. Antony Chen, Ro-Ting Lin

**Affiliations:** 1 Department of Epidemiology and Biostatistics, School of Public Health, University of Nevada, Las Vegas, Las Vegas, NV USA; 2 Department of Environmental and Occupational Health, School of Public Health, University of Nevada, Las Vegas, Las Vegas, NV USA; 3 Department of Occupational Safety and Health, College of Public Health, China Medical University, Taichung, Taiwan

**Keywords:** COVID-19, Temperature, Relative humidity, Precipitation, Lag, Spatial

## Abstract

**Background:**

The associations between meteorological factors and coronavirus disease 2019 (COVID-19) have been discussed globally; however, because of short study periods, the lack of considering lagged effects, and different study areas, results from the literature were diverse and even contradictory.

**Objective:**

The primary purpose of this study is to conduct more reliable research to evaluate the lagged meteorological impacts on COVID-19 incidence by considering a relatively long study period and diversified high-risk areas in the United States.

**Methods:**

This study adopted the distributed lagged nonlinear model with a spatial function to analyze COVID-19 incidence predicted by multiple meteorological measures from March to October of 2020 across 203 high-risk counties in the United States. The estimated spatial function was further smoothed within the entire continental United States by the biharmonic spline interpolation.

**Results:**

Our findings suggest that the maximum temperature, minimum relative humidity, and precipitation were the best meteorological predictors. Most significantly positive associations were found from 3 to 11 lagged days in lower levels of each selected meteorological factor. In particular, a significantly positive association appeared in minimum relative humidity higher than 88.36% at 5-day lag. The spatial analysis also shows excessive risks in the north-central United States.

**Significance:**

The research findings can contribute to the implementation of early warning surveillance of COVID-19 by using weather forecasting for up to two weeks in high-risk counties.

## Introduction

The coronavirus disease 2019 (COVID-19) became endemic in the United States in March 2020. California is the first state to implement the stay-at-home order on March 19 to curb the transmission of the disease [1]. Most of the other states issued either statewide or partial stay-at-home orders before April [2]. For a few states which never issued stay-at-home orders, like Arkansas or Iowa, most non-essential businesses still closed in those states with lockdown measures issued by cities or local governments [3].

Most state governments were facing tremendous pressure to loosen restrictions toward reopening. Even though some states still had not flattened the epidemic curve by May, public opinions expected that the pandemic could be mitigated along with the rise of temperature in summer months, just like the 2002–2004 outbreak of severe acute respiratory syndrome [4]. Laboratory experiments revealed that coronaviruses could better survive under a cool and dry ambient environment [5, 6]. Even though experts alerted that rising temperatures may not reduce the reproduction number of COVID-19 [7], the expectation of mitigating the COVID-19 pandemic in 2020 summer was accompanied by the suspicion of the continuance of the pandemic.

Several epidemiological studies about the association between COVID-19 and meteorology were published a few months after the outbreak. Studies in Indonesia and India indicated a significant association between temperature and COVID-19, but they did not find substantial evidence for relative humidity or rainfall [8, 9]. A 50-city study showed that countries with a lower temperature or humidity had a higher prevalence of COVID-19, while the morbidity decreased in countries during the summer when temperature increased above 30 °C [10]. A study in China implies that humidity is negatively associated with COVID-19 mortality, and particularly humidity plays a critical role in the relationship between air quality and COVID-19 mortality [11]. Another study investigating data from 100 cities in China during the early outbreak also stated that high temperature and high humidity reduced the reproduction number of COVID-19 [12]. However, other studies argued against such an assertion. For example, a study emphasized that, without immediate intervention, the number of COVID-19 cases could not decrease merely due to weather conditions [13]. A study in Spain concluded that temperature might not be a risk factor to induce COVID-19, but the rising temperature may suppress coronavirus transmissions [14]. Another similar study in China also suggested that higher temperature cannot slow down COVID-19 incidence [15].

The development of a meteorological factor-based model for disease forecast may contribute to public health strategic planning. Reviewing previous studies using data in the United States, we noticed diverse patterns between meteorological factors and infected cases with some differences possibly attributable to analytical methods. First, magnitudes and significant impacts of individual meteorological factors that influence the COVID-19 incidence remain inconclusive. An explicit example is that temperature may positively associate with COVID-19 [16] but may also not related to or even negatively related to COVID-19 [17, 18]. Using comparable spatiotemporal scales for meteorological and health data can resolve some of these contradictions [19]. Second, the literature has observed the lagged effect of meteorological factors or air pollution on COVID-19, but inconsistent time lags ranging from 1 to 21 days were applied [16–18, 20, 21].

To clarify how COVID-19 was affected by weather variabilities taking temporal trends into account, this study hypothesizes that the meteorological impacts on COVID-19 incidence may last for several days. This study’s primary purposes include evaluating the lagged effect of weather on COVID-19 for up to 2 weeks and determining the most influential meteorological factors. We surveyed more than 200 diverse counties across the United States to generalize our analytical findings. The objective is to elucidate the progress of the COVID-19 pandemic in the United States over a more extended period. This study can contribute to the development of early warning surveillance to project potential surges of COVID-19 incidence when unfavorable meteorological conditions were observed in advance.

## Methods

### Study areas and period

**Fig. 1:**
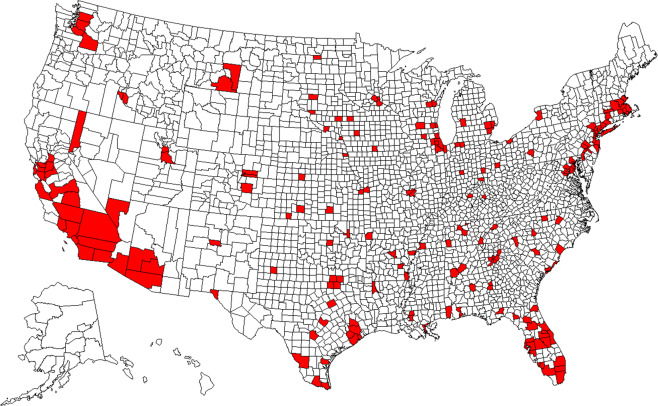
Top 203 high-risk counties of COVID-19 in the United States by October 31, 2020.

The study areas were determined to be high-risk counties, which contain (1) at least 10,000 accumulative confirmed cases of COVID-19 by October 31, 2020, and (2) at least 900 confirmed cases per 10,000 residents by October 31, 2020. The two criteria yielded 203 counties (including the District of Columbia) across 41 states where more counties (*N* = 21; % = 10.34) are located in Florida than in any other states (Fig. 1). The selected counties are representative because they contain 51.28% of the total population in the United States. In addition, this study included 78% of the top 200 counties with the most population. The study period was 10 months, from March 1 to October 31 in 2020.

### Data source

The daily accumulative confirmed cases of COVID-19 were downloaded from the New York Times public data repository (https://github.com/nytimes/covid-19-data). Here confirmed cases mean the number of individuals whose coronavirus infections were confirmed by a laboratory test that detects viral RNA in a sample. Daily new confirmed cases in each county were further calculated for time-series analysis. The meteorological data were based on long-term weather stations representative of each county, typically at the main airport, and downloaded from the Weather Underground historical archive (https://www.wunderground.com/history). Seven parameters were explored, including daily maximum temperature, minimum temperature, average temperature, maximum relative humidity, minimum relative humidity, average relative humidity, and precipitation. Those meteorological measures were used in a previous study conducted by the same authors [18]. This study also considered the following socioeconomic status measures at the county level from the American Community Survey’s 5-year (2014–2018) estimates [22]: median age, male percentage, White percentage, Black percentage, Hispanic percentage, at least high school education percentage, poverty percentage, no health insurance coverage percentage. The rationale for using those socioeconomic status measures is because they were ever used as confounding variables in the COVID-19 literature [18]. Lastly, a temporal indicator of statewide stay-at-home order was adopted from the New York Times [3]. We recorded 1 in the dates from the order issued to the order terminated and 0 in the other dates.

### Statistical analyses

We applied the distributed nonlinear lag model [23] to evaluate the nonlinear lagged impacts of weather variability on COVID-19. Assuming *Y*_*it*_ is the number of daily confirmed cases in county *i* at calendar time *t*, which follows a Poisson distribution with the mean parameter *μ*_*it*_, the model equation is:

$${\mathrm{log}}\left( {\mu _{it}} \right) =\, 	\alpha + f\left( {{\rm{TP}}_{it},{\rm{lag}}} \right) + f\left( {{\rm{RH}}_{it},{\rm{lag}}} \right) + f\left( {{\rm{PR}}_{it},{\rm{lag}}} \right) + {\boldsymbol{\beta}} \left( {{\rm{State}}_i} \right)\\ 	+ {\boldsymbol{\gamma}} \left( {{\rm{DOW}}_t} \right) + {\boldsymbol{\delta}} \left( {{\rm{Confounder}}_i} \right) + f\left( t \right) + f\left( {{\rm{lon}}_i,{\rm{lat}}_i} \right) + {\mathrm{offset}}$$

where TP_*it*_, RH_*it*_, and *PR*_*it*_ are the three main meteorological factors for temperature, relative humidity, and precipitation, respectively. All of them were analyzed by a cross-basis function equivalent to an interaction between a meteorological basis function and a lag basis function. Both basis functions were set to the natural cubic B-spline, and the maximum lag was set to 14 days to consider the incubation period of COVID-19 [24]. The linear vectors State_*i*_ and DOW_*t*_ were appended to take into account state heterogeneity and the short-term day-of-the-week autocorrelation. The linear vector Confounder_*i*_ considered confounding effects from socioeconomic status measures and stay-at-home order. The slopes of the three vectors were denoted by ***β***, ***γ***, and ***δ***, respectively. The term *f*(*t*) is a natural cubic spline for the calendar time variable *t* with 8 degrees of freedom to control long-term autocorrelations. The term *f*(lon_*i*_, lat_*i*_) is a two-dimensional spatial function for county’s coordinate in longitude and latitude to control spatial autocorrelations. The last term is an offset from the logarithm of the county population, according to the latest 2018 population estimate released by the American Community Survey.

Because either temperature or relative humidity has three measures (i.e., minimum, average, and maximum), we only considered one measure in the model. We determined the best combination from maximum temperature and minimum relative humidity with the smallest quasi-Akaike information criteria in the final model by conducting a model selection. We further selected the degrees of freedom in each basis function. Here we defined that the basis function of lag has the same number of knots, placing at equally spaced values of the logarithmic scale of lag to allow flexibility in the first part of the distributed curve where higher flexibility is expected. The knot selection in the lag basis function was chosen from four to eight knots when all meteorological basis function has the same number of knots. The smallest quasi-Akaike information criteria appeared the best number of knots by eight knots on the lag basis function. Finally, based on the eight knots in the lag basis function, we determined the best number of knots in the maximum temperature (four knots), minimum relative humidity (six knots), and precipitation (four knots).

Each estimated coefficient was transformed into a relative risk (RR) or an increased percentage of relative risk (RR%). In the cross-basis function, the reference levels of all meteorological and lag variables were 0 s. The estimated spatial function was further smoothed within the entire continental United States using the biharmonic spline interpolation [25]. Sensitivity analyses were performed in the best model to see whether different lengths of lag may affect the result. We considered 12-day and 16-day lags to see whether each cross-basis function’s estimated coefficients were similar to those from 14-day lags by using the mean square error.

Data cleaning and management were performed in SAS v9.4 (SAS Institute, Inc., Cary, NC, USA). Data analyses and visualization were performed in RStudio v1.3.1093 (RStudio, Inc., Boston, MA, USA). Biharmonic spline interpolation and mapping were conducted by Matlab R12 (The MathWorks, Inc., Natick, Massachusetts). The significance level was set to 0.05.

## Results

Table 1 shows that, on average, there were 102.39 daily new confirmed cases per county (standard deviation = 226.91), where the maximum was 14,129 cases, appearing in Harris County, Texas, on September 21, 2020. The maximum daily temperature reached as high as 121 °F, occurring in Riverside County, California. Because the study period is long enough, the minimum relative humidity ranged from 0 to 100%, while the average was 43.70% (Standard deviation = 18.63). The distribution of precipitation was right-skewed as over 75% of observations were <0.25 mm. The highest daily precipitation was 202.44 mm, observed in two counties of Louisiana: East Baton Rouge County and East Felici County.

**Table 1:** Summary statistics of COVID-19 and the selected meteorological measures from March to October in 2020 among 203 counties in the United States.

	Mean	SD	Min	Q1	Q3	Max
New confirmed case	102.39	226.91	0.00	8.00	109.00	14,129.00
Maximum temperature (°F)	77.60	14.98	0.00	68.00	89.00	121.00
Minimum relative humidity (%)	43.70	18.63	0.00	31.00	57.00	100.00
Precipitation (mm)	2.58	8.70	0.00	0.00	0.25	202.44

*SD* standard deviation, *Q1* 1st quartile, *Q3* 3rd quartile.

**Fig. 2:**
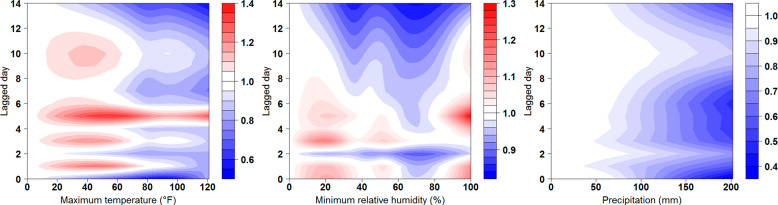
Relative risks of COVID-19 incidence varied by measurements and lagged days of the maximum temperature, minimum relative humidity, and precipitation from March 1 to October 31 in 2020 in the United States.

Figure 2 shows a wide range of RR on COVID-19 for the three selected meteorological variables and by lagged days. Higher RRs more likely happened in the maximum temperature between 20 and 120 °F from lag 4–6. The highest RR was 1.35 (95% confidence interval [CI] = 0.98, 1.85) at 55 °F and lag 5. Moreover, higher RRs more likely appeared in the minimum relative humidity <40% and more than 80% from lag 3 to 6. The highest RR was 1.30 (95% CI = 1.11, 1.51) at 100% and lag 5. Lastly, for the precipitation, most RRs were <1, especially when it was over 100 mm from lag 0 to 14.

**Fig. 3:**
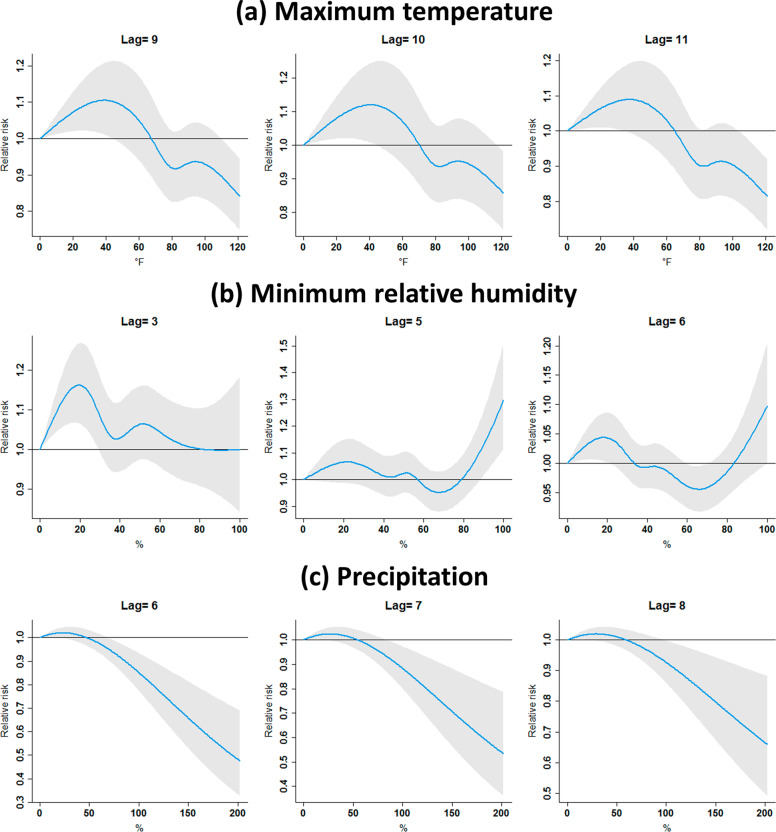
Relative risks of COVID-19 incidence at selected lagged days. **a** maximum temperature, **b** minimum relative humidity, and **c** precipitation at selected lagged days. Shaded areas are 95% confidence intervals.

Figure 3 depicts detailed variations of RR for each meteorological factor at selected lagged days where RRs were significantly larger than 1. Figure 3a shows that the maximum temperature levels lower than 44.40, 44.02, and 34.96 °F were significantly positively associated with COVID-19 incidence at lag 9, lag 10, and lag 11, respectively. Lower minimum relative humidity was also positively related to COVID-19 incidence at lag 3 and lag 6. In comparison, another significantly positive association was found when the minimum relative humidity was higher than 88.36% at lag 5, shown in Fig. 3b. Lastly, Fig. 3c presents that the precipitation levels lower than 18.91, 22.17, 25.08 mm were significantly positively associated with COVID-19 incidence at lag 6, lag 7, and lag 8, respectively.

**Fig. 4:**
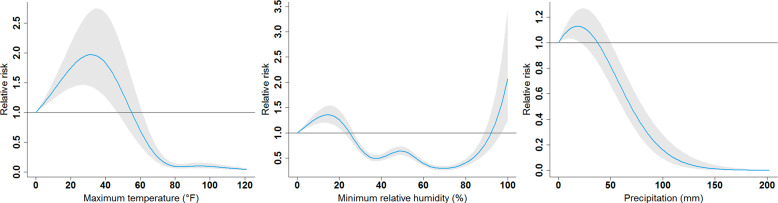
Overall impacts of the maximum temperature, minimum relative humidity, and precipitation on the relative risks of COVID-19 incidence. Shaded areas are 95% confidence intervals.

Figure 4 presents the overall impact of each selected meteorological factor after accumulating the RRs across all lags. When the maximum temperature increased from 0 °F, the RR started rising from 1 and reached the highest level (RR = 1.97; 95% CI = 1.43, 2.71) at 31.79 °F. After getting to the highest level, the RR started decreasing and consistently went below 1 as the maximum temperature elevated more than 55.07 °F. When the maximum temperature increased over 61.17 °F, the RR became significantly <1 through 121 °F. Moreover, the minimum relative humidity had the RR significantly larger than 1 when it was lower than 22.40% or higher than 97.02%. The highest RR was 2.07 (95% CI = 1.24, 3.46) when the minimum relative humidity reached 100%. Furthermore, similar to the maximum temperature, the precipitation also had a rising RR from 0 mm and achieved the highest RR of 1.13 (95% CI = 1.02, 1.25) at 18.46 mm. Afterward, the RR started decreasing to significantly below 1 when the precipitation was higher than 50.26 mm.

Among linear confounding variables, except the percentages of poverty and no health insurance coverage, all the others were significantly associated with COVID-19 incidence. Among socioeconomic status, male percentage had the highest RR% of 4.23% (*p* value < 0.0001; 95% CI = 2.43, 6.07). By defining Alabama and Sunday as the reference levels, Oklahoma and Friday had the highest RR by 2.56 (*p* value <0.0001; 95% CI = 1.69, 3.86) and 1.29 (*p* value <0.0001; 95% CI = 1.25, 1.34), respectively. One can refer to Tables S1 and S2 in Appendix for the results of the other confounding variables.

**Fig. 5:**
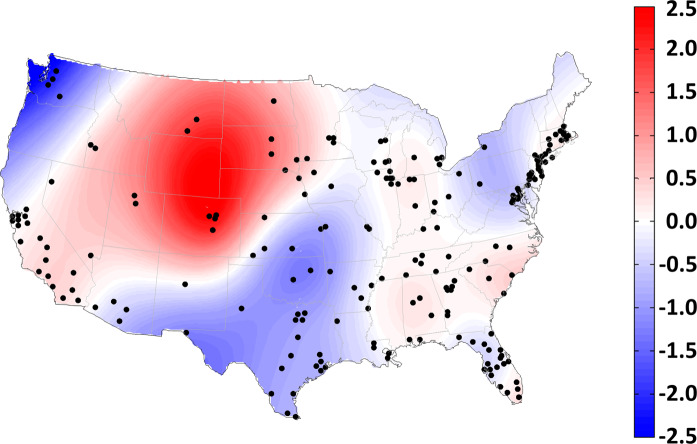
Smoothed spatial estimates on COVID-19 relative risk. Black dots represent the centroids of 203 counties.

The estimated spatial function was used to compute the RR of COVID-19 at the county level, as shown in Fig. S1 in Appendix. After controlling all linear and nonlinear effects, 7.88% (*N* = 16) of the counties had a significantly RR larger than 1, and 21.18% (*N* = 43) of the counties had a significantly RR smaller than 1. The largest RR was found in Denver County, Colorado (RR = 12.32; 95% CI = 5.64, 26.90). After smoothing the estimated spatial function to interpolate spatial estimates over 48 contiguous states, Fig. 5 reveals that most regions still had an excessive risk of COVID-19, except the northwestern states (e.g., Washington and Oregon), southern states (e.g., Oklahoma, Texas, Louisiana, and New Mexico), and northeastern states (e.g., New York, Pennsylvania, and West Virginia). In particular, higher excessive risks appeared in several north-central states, such as Montana, Wyoming, Colorado, North Dakota, and South Dakota.

Sensitivity analyses with the length of lag becoming 12 and 16 resulted in similar estimated coefficients. Comparing between the models using 12 and 14 lags, mean square errors of the three cross-basis functions were 0.0034, 0.0004, and 0.0003. Comparing between the models using 14 and 16 lags, mean square errors of the three cross-basis functions were 0.0041, 0.0003, and 0.0032.

## Discussion

Among diverse environmental triggers of COVID-19, meteorological factors were regarded as important predictors from the beginning of the pandemic because COVID-19 is similar to other respiratory diseases, which are generally associated with weather variability. COVID-19 can be more sensitive to weather conditions than respiratory infections such as influenza due to the potential aerosol route of transmission, in addition to person-to-person transmissions through droplets or contact [26, 27]. Through the route, SARS-CoV-2 attached to the aerosol surface can remain infectious for a longer range of transport in the air. However, COVID-19 research rarely considered the lagged effect resulting from the incubation period or asymptomatic carriers, compared to the literature of respiratory diseases [28, 29]. This study has an apparent assumption: the time that people who were confirmed positive with COVID-19 is not exactly the time meteorological factors triggered the infection. We found that maximum temperature, minimum relative humidity, and precipitation better explained COVID-19 incidence than the other meteorological factors through a series of advanced analyses. Moreover, a higher risk may happen at lower maximum temperature and higher minimum relative humidity with a shorter lag. Furthermore, a significantly positive association was more likely observed between COVID-19 and lower precipitation levels when considering a longer lag. Lastly, the spatial analysis found that counties located in the north-central states had an excessive risk of COVID-19, which implies that counties located in those states may still have unobserved triggers elevating the COVID-19 incidence.

The literature examined the lagged effect of meteorological factors on virus-communicable diseases from <7 days to over 1 month. For example, temperature or circulation weather types may have lagged effects on influenza from 2 to 7 days [30–32]. There could be a longer lagged effect for measles up to 30 days because of the relative humidity [33]. Zika virus infections can even experience the lagged effect lasting 20 weeks at most [34]. Compared to these virus-communicable diseases, the length of lagged effect for COVID-19 is only up to 2 weeks because its incubation period is around 14 days. That is why the Centers for Disease Control and Prevention recommends the 14-day quarantine for travels or following exposure to infected persons [35], even though it is still debatable [36, 37]. In particular, COVID-19 vaccines have been deployed in the United States since December of 2020. As more and more people were vaccinated, whether the period of lagged effect on COVID-19 will become longer needs further assessments.

Population-based studies have investigated the association between meteorological factors and COVID-19 globally, but the lagged effects were not examined [8, 38], only covered for fewer lags [20, 39], or assumed for linear impacts [8, 40]. Despite some diversities in model settings among relevant research, most findings were similar in that escalated COVID-19 risk was more associated with lower temperature and humidity levels. A debatable finding here might be the overall positive impact of higher minimum relative humidity levels because a negative association between humidity and COVID-19 is more supported by literature [12, 41, 42]. Our finding is partially consistent with another research using data from eight cities in the United States [21], as both studies use the same modeling approach, which shows lasting effects of temperature and humidity on the COVID-19 incidence. Nonetheless, the 8-city study only investigated a lag period of 7 days and did not examine precipitation. Some inconsistent results came from using other temperature and humidity measures, such as the diurnal temperature range, wind speed, or absolute humidity [43, 44].

Compared to temperature and humidity, the precipitation impact on COVID-19 was less discussed because precipitation was either used as a confounding variable [45–47] or not significantly associated with COVID-19 [48]. A few studies mentioned that precipitation might increase COVID-19 incidence while only examining linear associations [49, 50]. This study built a nonlinear model to suggest that positive associations only occurred when the precipitation level was <50.26 mm. Nonetheless, areas with a higher precipitation level may still suffer from COVID-19 because of a higher humidity level. In addition, the spatial analysis showed that, among the top 10 counties with the most excessive risk, 4 of them are located in Colorado. An earlier study examined the geographic patterns of COVID-19 during an initial period from March 11 to April 8 revealed several hot spots in Denver and nearby counties [51], which are consistent with our findings. Their findings also found that asthma hospitalization had a potential spatial overlap with COVID-19 incidence, while asthma hospitalization was not considered a predictor in this study. Hence, it is essential to have a spatial function to catch unobserved predictors’ influence in our model. A GIS-based spatial analysis explored the relationship between environmental, socioeconomic, and demographic variables and COVID-19 [52] to conclude a higher association between COVID-19 incidence and the percentage of nurse practitioners in the central and midwestern states, which is consistent with our spatial findings. Further research in those hot spot counties is needed, especially on assessing unobserved triggers like air pollution [53] or social distancing [54] with a more extended study period.

This research’s strength is that the study period is longer than other similar studies to capture the weather variability [21, 39, 55]; thus, the results shall be more reliable. The statistical model also included a spatial function, which can take unobserved triggers into account and optimize the model-fitting to elucidate risky areas from other counties not selected in this study. However, there are still a few limitations. First, the so-called “high-risk” counties came from self-definition. Even though this study adopted two criteria (i.e., accumulative confirmed cases and crude rates), there is no concrete standard to determine whether a county is at a high risk of COVID-19. Analyzing all counties (>3000) is more objective, but the total sample size will inflate to 0.76 million, causing an extremely intensive computation when applying such an advanced and complicated modeling approach. Hence, our findings may not apply to counties at a lower risk of COVID-19 in the United States. Second, the DLNM cannot estimate county-specific meteorological impacts like a mixed model because the cross-basis function cannot vary by county. The literature has shown that temperature and humidity may influence COVID-19 diversely in different areas of the same country [21, 56]. To further study the relationship between meteorological factors and COVID-19 in counties of interest, the DLNM may be used to analyze a specific county rather than pooling all counties together. However, selecting the best meteorological factors for each county based on a systematic model selection procedure will be tedious.

## Conclusions

This study concludes that lower maximum temperature, minimum relative humidity, and precipitation posed higher risks to COVID-19 incidence up to 14 days in the United States. Our findings can contribute to early warning surveillance of COVID-19 when weather forecasting predicts lower temperature or relative humidity coming within 2 weeks. The spatial analysis reveals potentially excessive risk areas that meteorological factors cannot fully explain, and the result demonstrates other unobserved risk factors in hot spot areas. Our findings emphasize the need for further aggressive interventions to curb the surge of COVID-19 incidence in high-risk counties.

## Supplementary information

Supplementary Information

## Data Availability

All data used in this study are going to be submitted to Kaggle upon acceptance of the manuscript.
