# Supplementary material for: Lagged meteorological impacts on COVID-19 incidence among high-risk counties in the United States—a spatiotemporal analysis
Source: J Expo Sci Environ Epidemiol. 2021 Jul 1;32(5):774–81. doi: 10.1038/s41370-021-00356-y (PMC8247626; doi:10.1038/s41370-021-00356-y)

**Appendix**

Table S1. Associations between confounding variables (socioeconomic status, stay-at-home order, and the day-of-week) and COVID-19.

| **Variable** | **RR%** | **95% CI** | | **P-value** |
| --- | --- | --- | --- | --- |
| Median age | 0.87 | 0.21 | 1.54 | 0.0088 |
| Male percentage | 4.23 | 2.43 | 6.07 | <0.0001 |
| White percentage | -0.90 | -1.30 | -0.51 | <0.0001 |
| Black percentage | 0.28 | 0.07 | 0.49 | 0.0075 |
| Hispanic percentage | 1.03 | 0.66 | 1.40 | <0.0001 |
| At least high school education percentage | -2.17 | -2.76 | -1.59 | <0.0001 |
| Poverty percentage | 0.53 | -0.01 | 1.08 | 0.0537 |
| No health insurance coverage percentage | 0.69 | -0.11 | 1.50 | 0.0894 |
|  | **RR** | **95% CI** | | **P-value** |
| Stay-at-home order | 1.70 | 1.61 | 1.80 | <0.0001 |
| Monday | 1.05 | 1.01 | 1.08 | 0.0166 |
| Tuesday | 1.08 | 1.04 | 1.12 | <0.0001 |
| Wednesday | 1.13 | 1.09 | 1.17 | <0.0001 |
| Thursday | 1.21 | 1.17 | 1.25 | <0.0001 |
| Friday | 1.29 | 1.25 | 1.34 | <0.0001 |
| Saturday | 1.15 | 1.11 | 1.19 | <0.0001 |

Abbreviation: RR% = Relative risk percentage; CI = Confidence interval

Table S2. Associations between state confounding variables and COVID-19.

| **State^†^** | **RR**^††^ | **95% CI** | | **P-value** |
| --- | --- | --- | --- | --- |
| Arizona | 0.68 | 0.34 | 1.36 | 0.2760 |
| Arkansas | 1.94 | 1.40 | 2.68 | 0.0001 |
| California | 0.16 | 0.07 | 0.36 | <0.0001 |
| Colorado | 0.02 | 0.01 | 0.05 | <0.0001 |
| Connecticut | 0.29 | 0.16 | 0.50 | <0.0001 |
| Delaware | 0.67 | 0.39 | 1.15 | 0.1456 |
| District of Columbia | 1.09 | 0.63 | 1.87 | 0.7643 |
| Florida | 1.39 | 1.13 | 1.70 | 0.0015 |
| Georgia | 0.78 | 0.66 | 0.93 | 0.0046 |
| Idaho | 0.25 | 0.10 | 0.62 | 0.0024 |
| Illinois | 0.40 | 0.23 | 0.69 | 0.0011 |
| Indiana | 0.41 | 0.24 | 0.71 | 0.0013 |
| Iowa | 0.92 | 0.51 | 1.67 | 0.7831 |
| Kansas | 1.41 | 0.90 | 2.22 | 0.1342 |
| Kentucky | 0.66 | 0.43 | 1.03 | 0.0635 |
| Louisiana | 1.52 | 1.17 | 1.99 | 0.0018 |
| Maryland | 0.99 | 0.59 | 1.67 | 0.9702 |
| Massachusetts | 0.30 | 0.17 | 0.55 | 0.0001 |
| Michigan | 0.30 | 0.17 | 0.54 | 0.0000 |
| Minnesota | 0.33 | 0.15 | 0.71 | 0.0045 |
| Missouri | 0.94 | 0.61 | 1.46 | 0.7959 |
| Montana | 0.11 | 0.02 | 0.57 | 0.0083 |
| Nebraska | 0.69 | 0.37 | 1.27 | 0.2245 |
| Nevada | 0.30 | 0.14 | 0.68 | 0.0033 |
| New Jersey | 0.43 | 0.26 | 0.72 | 0.0013 |
| New Mexico | 0.19 | 0.11 | 0.33 | <0.0001 |
| New York | 0.46 | 0.27 | 0.78 | 0.0037 |
| North Carolina | 0.52 | 0.37 | 0.74 | 0.0002 |
| North Dakota | 0.57 | 0.08 | 3.81 | 0.5566 |
| Ohio | 0.43 | 0.26 | 0.73 | 0.0016 |
| Oklahoma | 2.56 | 1.69 | 3.86 | <0.0001 |
| Pennsylvania | 0.50 | 0.30 | 0.83 | 0.0064 |
| Rhode Island | 0.41 | 0.22 | 0.74 | 0.0026 |
| South Carolina | 0.89 | 0.68 | 1.17 | 0.4048 |
| South Dakota | 0.48 | 0.21 | 1.10 | 0.0817 |
| Tennessee | 0.96 | 0.77 | 1.19 | 0.6890 |
| Texas | 1.75 | 1.15 | 2.65 | 0.0080 |
| Utah | 0.13 | 0.05 | 0.31 | <0.0001 |
| Virginia | 0.83 | 0.50 | 1.39 | 0.4746 |
| Washington | 1.37 | 0.45 | 4.18 | 0.5798 |
| Wisconsin | 0.54 | 0.31 | 0.96 | 0.0352 |

Abbreviation: RR = Relative risk; CI = Confidence interval
^†^ Only 40 states were included because no county was selected in the following states: Alaska, Hawaii, Maine, Mississippi, New Hampshire, Oregon, Vermont, West Virginia, Wyoming  ^††^ The reference level is Alabama.

Figure S1. Relative risks of COVID-19 incidence and 95% confidence interval derived from the estimated spatial function.


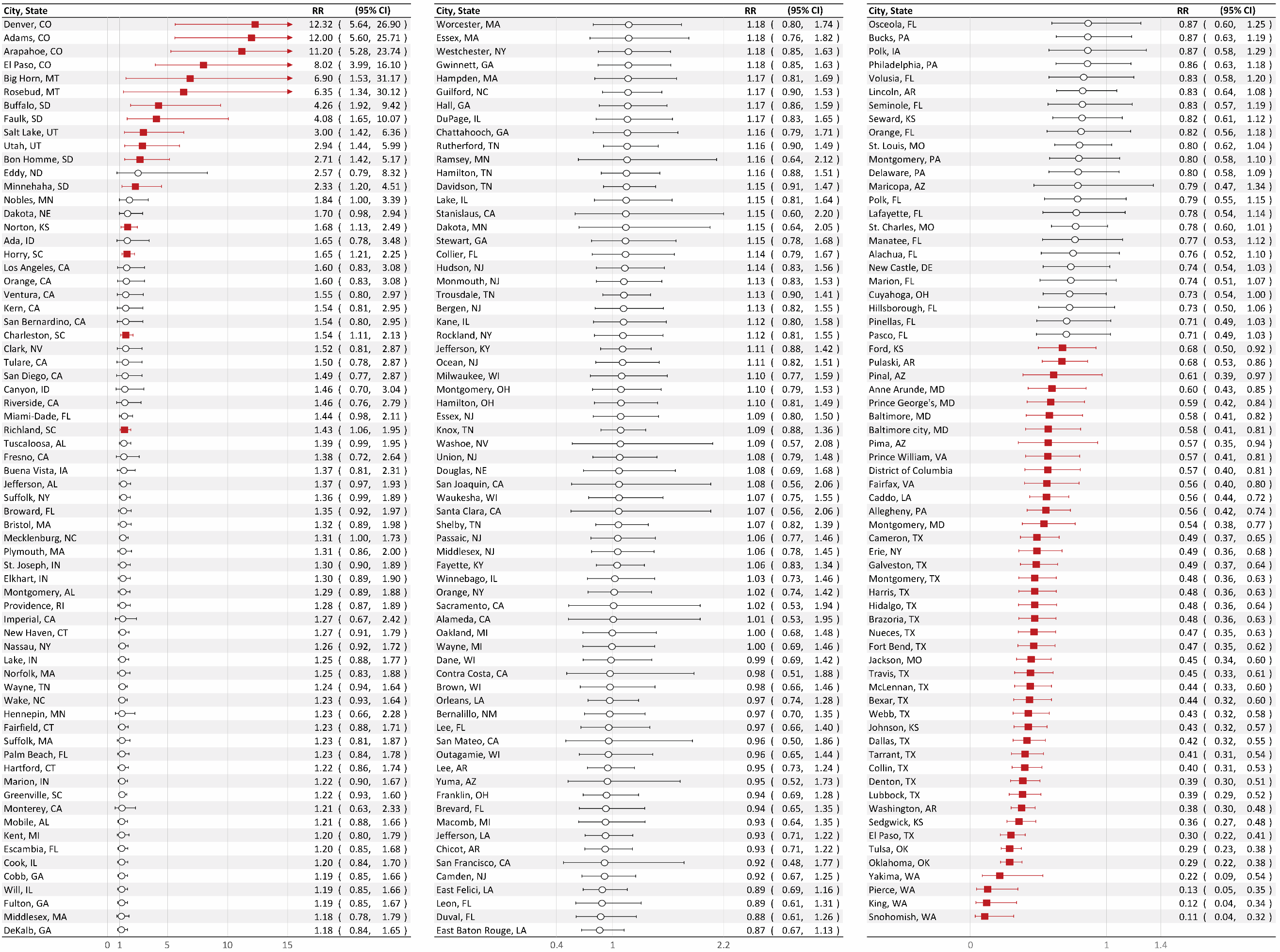

Supplement: Supplementary file 1 — Supplementary Information [file 41370_2021_356_MOESM1_ESM.docx]
